# Disease-Modifying Symptomatic Treatment (DMST): The Potential Role of Vortioxetine in the Treatment of Depression in Patients with Multiple Sclerosis

**DOI:** 10.2174/011570159X326862240909105845

**Published:** 2024-09-11

**Authors:** Ettore Dolcetti, Pietro Annovazzi, Marinella Clerico, Eleonora Cocco, Antonella Conte, Girolama Alessandra Marfia, Marco Salvetti, Valentina Tomassini, Valentina Torri Clerici, Rocco Totaro, Antonio Bruno, Diego Centonze

**Affiliations:** 1 IRCCS Neuromed, Pozzilli (IS), Italy;; 2 Neuroimmunology Unit - Multiple Sclerosis Centre ASST Valle Olona - Gallarate Hospital, Gallarate (VA), Lombardia, Italy;; 3 Clinical and Biological Sciences Department, University of Torino, Torino, Italy;; 4 Department of Medical Science and Public Health, Centro Sclerosi Multipla, University of Cagliari, Cagliari, Italy;; 5 Department of Human Neurosciences, Sapienza University of Rome, Rome, Italy;; 6 Department of Systems Medicine, Tor Vergata University, Rome, Italy;; 7 Multiple Sclerosis Clinical and Research Unit, Tor Vergata University Hospital, Rome, Italy;; 8 Centre for Experimental Neurological Therapies (CENTERS), Department of Neurosciences, Mental Health and Sensory Organs, Sapienza University of Rome, Rome, Italy;; 9 Institute of Advanced Biomedical Technologies (ITAB), Department of Neurosciences, Imaging and Clinical Sciences, University G. d’Annunzio of Chieti-Pescara, Chieti, Italy;; 10 Neuroimmunology Unit, IRCCS Istituto Neurologico C. Besta, Milan, Italy;; 11 Demyelinating Disease Center, Department of Neurology, San Salvatore Hospital, L’Aquila, Italy

**Keywords:** Vortioxetine, multiple sclerosis, cognition, neuroinflammation, depression, disease-modifying treatment

## Abstract

In multiple sclerosis (MS), alongside the physical symptoms, individuals often grapple with anxiety and depressive symptoms as prevalent comorbidity. Mood disturbances, frequently undertreated in clinical practice, significantly impact the quality of life of individuals with MS, exacerbating disability and hindering overall well-being. Furthermore, traditional antidepressant therapies are often associated with adverse events, such as sexual side effect, weight gain, which could limit their use in these patients. Vortioxetine is one of the most innovative antidepressant drugs in the current pharmacopeia. Its pharmacological profile includes serotonin reuptake inhibition, antagonism for hydroxytryptamine (HT) receptors 5-HT3, 5-HT1D and 5-HT7, partial agonism for 5-HT1B, and agonism for 5-HT1A. It has been shown to have a beneficial effect on depression-related cognitive dysfunction, as well as on anxiety, depression, anhedonia and emotional blunting. Recently a potential anti-inflammatory action was also described. Limited clinical studies have specifically explored the efficacy of vortioxetine in treating depressive symptoms in MS. However, extrapolating from existing research in major depressive disorder, it is plausible that vortioxetine's multimodal mechanism could provide a favorable therapeutic approach. This position paper, which summarizes the output of annual clinical meeting held by the DMSTs in MS Italian Study Group, is focused on the possible role that vortioxetine could play as symptomatic treatment (ST) of depressed patients with MS, hypothesizing a direct impact on the clinical course of the disease.

## 1 INTRODUCTION

### 1.1 The Concept of Disease-modifying Symptomatic Treatment (DMST) in Multiple Sclerosis

The drugs approved for the treatment of multiple sclerosis (MS) are classified into therapies capable of modifying the course of the disease (disease-modifying therapies, DMTs) and symptomatic therapies (STs). While DMTs and STs are commonly believed to target distinct pathophysiological aspects of MS, evidence exists that their division into two classes is somehow artificial, as both DMTs and STs intervene to some extent on pathophysiology and symptom control in MS, influencing each other’s effects. Optimizing MS treatment by closing the divide between DMTs and STs is the specific aim of the DMSTs in MS Italian Study Group, composed of MS specialists with long-standing clinical experience in the management of the disease and regularly meeting to discuss the DMST potential of specific pharmacological and non-pharmacological interventions.

## 2 DEPRESSIVE SYMPTOMS IN MS: A DISORDER OF THE IMMUNE SYSTEM

Mood disorders, and in particular depressive symptoms, represent a relevant aspect of the clinical presentation of MS, affecting young individuals and often underrecognized and undertreated [1, 2]. Their prevalence is interestingly similar in men and women with MS, although the clinical presentation of the disease can be different [3]. Depressive symptoms in MS patients have been historically interpreted as the result of the reasonable emotional reaction to the diagnosis and to physical limitations associated with the later stages of MS, often of the damage of specific brain areas [4-6], black holes, and brain atrophy [7]. Other studies found a relationship between depression and the presence of active lesions at the magnetic resonance imaging (MRI), independently of the lesion site, and an association between suicidal ideation and elevated proinflammatory cytokine levels [8, 9]. Evidence suggests that depressive symptoms in MS could be largely mediated by some of the same processes involved in the immunopathogenesis of this neurologic disease. In particular, the increase in proinflammatory cytokines, activation of the hypothalamic-pituitary-adrenal (HPA) axis, and reduction in neurotrophic factors, such as monoaminergic and glutamatergic transmission increased the rate of depression seen in MS [10-12]. In MDD, monoaminergic and neuroendocrine hypotheses are likely to have greater significance, while in MS, neuroinflammatory and glutamatergic theories prevail [12]. The incidence of mood disorders in MS appears to be twice as high as in the general population [13, 14], with a prevalence variable from 24% to 54% and a higher frequency than in other neurological diseases, equally disabling but lacking underlying inflammatory mechanisms [15]. A similar prevalence of mood disorders was found in MS and chronic bowel disease, emphasizing the role of inflammation in mood control [15]. It is also known that the use of some immunosuppressive drugs in autoimmune diseases improves symptoms related to a concomitant mood disorder [16]. The physical clinical features of MS influence the phenotype and severity of depressive symptoms and vice versa. In fact, an association was found between the severity of depressive symptoms, measured by the BDI score, and disability assessed using the EDSS [17]. Higher BDI and STAI scores during radiological reactivation and increased STAI score in patients 6 months before experiencing MS relapses supports the conclusion that depression could not be interpreted as a reactive phenomenon [17]. Differences in the presence of comorbid depression exist among the different phenotypes of MS: a phenotype common to sickness behavior, characterized by the prevalence of acute inflammation, is typical in relapsing-remitting (RR-MS) forms [18, 19], while a more pronounced cognitive and attentional involvement, less responsive to conventional therapies, is more characteristic of progressive forms (P-MS) of disease, due to microglial activation and consequent neurodegeneration [19].

## 3 VORTIOXETINE: MECHANISMS OF ACTION AND SPECIFICAL CLINICAL EFFECT

**Fig. (1):**
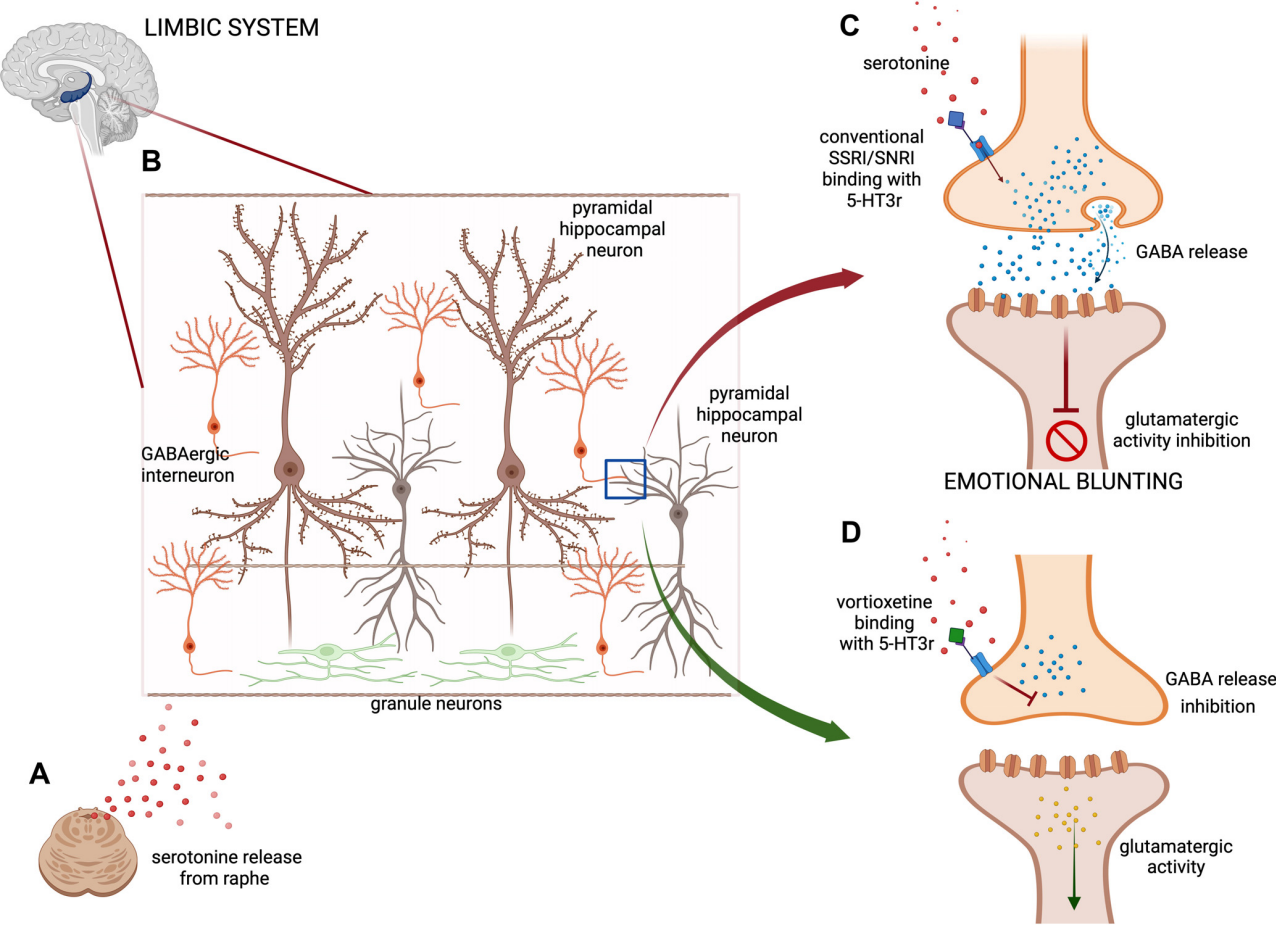
Effects of vortioxetine in the regulation of GABAergic and glutamatergic transmission in the limbic system and hippocampus. (**A**) Release of serotonin influences GABAergic interneuron activity in the limbic system and hippocampus (**B**). (**C**) In the presence of conventional SSRI/SNRI, serotonin binds, activating 5-HT3, and GABA is released from the presynaptic terminal, inhibiting glutamatergic activity in the post-synaptic terminal of pyramidal neurons and establishing the biochemical basis of emotional blunting. (**D**) Vortioxetine, acting as a 5-HT3 antagonist, inhibits GABA release from the presynaptic terminal, thus favoring glutamatergic activity in the pyramidal neurons. **Abbreviations**: GABA (gamma-aminobutyric acid); SSRI (selective serotonin reuptake inhibitor); 5HT-3 (5 hydroxytryptamine-3); 5HT-3r (5 hydroxytryptamine-3 receptor).

Among antidepressant drugs, vortioxetine represents one of the most impactful and innovative molecules in the pharmacological treatment of mood disorders [20] with a multimodal action. Its pharmacological profile includes inhibition of the serotonin transporter and modulation of serotonin receptors with a specific antagonism for hydroxytryptamine (HT) receptors 5-HT3, 5-HT1D, and 5-HT7, partial agonism for 5-HT1B, and agonism for 5-HT1A. Preclinical studies suggest neuromodulation of multiple systems, including 5-HT, NE, dopamine, acetylcholine, histamine, glutamate, and gamma-aminobutyric acid (GABA) systems in cortical and hippocampal neurons [21]. Indeed, traditional antidepressants, such as selective serotonin reuptake inhibitors and serotonin (SSRI) and noradrenaline reuptake inhibitors (SNRI), showed a limited benefit on anhedonia [22], with scattered evidence of efficacy in this direction for new antidepressant drugs, such as agomelatine [23-27], and ketamine [28, 29]. Emotional blunting has been proposed to relate to serotonergic effects in the frontal lobes by the projection of the midbrain dopaminergic reservoir to the prefrontal cortex [30]. By broadly enhancing serotoninergic transmission, SSRI and SNRI drugs activate gamma-aminobutyric acid (GABA) interneurons, dampening the noradrenergic as well as the dopaminergic input [31] (Figs. **1A**-**D**). The mechanism by which vortioxetine is efficacious in adults with MDD who experience emotional blunting is not fully understood, but its action as a 5HT3 receptor antagonist seems to modulate gamma-aminobutyric acid (GABA) interneurons, preventing glutamatergic impairment. (Figs. **1A**-**D**). Patients receiving vortioxetine improved emotional blunting, with amelioration of overall functioning, motivation and energy, cognitive performance, and depressive symptoms in patients with MDD with partial response to SSRI/SNRI therapy [32], also in elderly people [33-37]. A network meta-analysis in patients with MDD indicated that vortioxetine was the only antidepressant that exerted statistically significant effects on the Digit Symbol Substitution Test (DSST) between baseline and follow-up when compared with both placebo and all other antidepressants analyzed [38]. Vortioxetine also has an adequate safety profile compared to other antidepressant drugs, particularly in terms of weight gain, sexual dysfunction, and safety in terms of no QT prolongation with the absence of pharmacokinetic interactions on P450 isoenzyme [34, 39]. Recent evidence shows a role of vortioxetine also in the treatment of chronic neuropathic pain (CNP), mediated by 5-HT3 postsynaptic antagonism that causes reduction of hyperalgesia and 5-HT7 postsynaptic antagonism increasing analgesia [40]. Vortioxetine seems to show superiority at low dosages compared with conventional SNRIs (duloxetine and venlafaxine) both in animal models [41, 42] and in human studies [40, 43]. An increase of BDNF levels mediated by vortioxetine could act in the recovery from chronic pain, restoring neuronal connection and acting on synaptic plasticity [44, 45]. Vortioxetine is characterized by a multidimensional profile, including action on both depressive symptoms and comorbid major depression in patients with MS, with different neurobiological underpinnings, among which amelioration of cognitive symptoms [46, 47]. Potential anti-inflammatory properties observed in preclinical studies [48, 49], as well as interesting efficacy and tolerability results of clinical studies with specific depressed neurological patients such as patients with Parkinson's disease or Alzheimer's disease [50, 51], make vortioxetine a promising therapeutic option for patients with MDD and concomitant conditions, as MS.

## 4 TREATMENT OF DEPRESSION IN MS: EFFECTS OF VORTIOXETINE ON FATIGUE AND COGNITION

Cognitive deficits are commonly observed in MS [52, 53], and several studies showed that vortioxetine can positively impact cognitive impairment during depressive episodes [33]. Vortioxetine also showed efficacy in the treatment of MDD, including those with a high level of anxiety symptoms, in MDD-associated physical symptoms [54], and in depressed patients with neurodegenerative disorders in comorbidity, such as Alzheimer's disease [55, 56] and Parkinson’s disease (Table **1**) [50]. Together, these data support the idea that this drug could potentially ameliorate the cognitive deficits and fatigue frequently encountered also in MS patients. In this respect, data available in the literature are few and refer to a recent small study conducted on 17 depressed MS patients, in which the authors demonstrated a significant amelioration with vortioxetine administration in health status, anxiety, and general health test for the vitality domains, with no significative association for attention, information processing speed, and fatigue, probably due to a low population size [57]. Therefore, larger studies are needed in this direction.

## 5 IMMUNOMODULATORY EFFECTS OF VORTIOXETINE

**Fig. (2):**
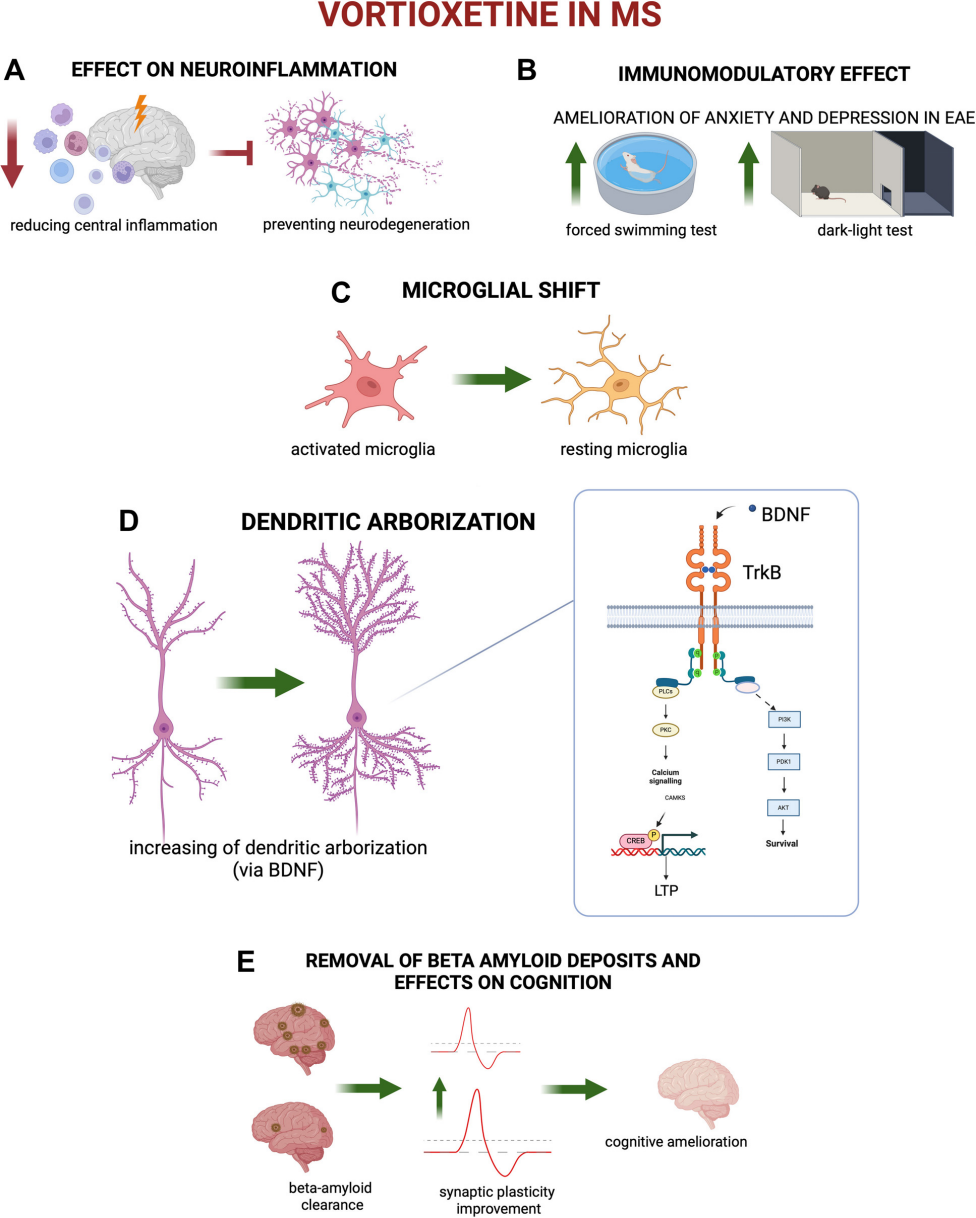
Effects of Vortioxetine in depressed patients with MS. (**A**) Vortioxetine seems to influence neuroinflammation in MS, acting as an anti-inflammatory drug and preventing neurodegeneration. (**B**) Vortioxetine exerts an immunomodulatory effect by ameliorating the anxiety (light dark test) and depressive (forced swimming test) clinical phenotype in EAE mice. (**C**) Vortioxetine induces microglia shift from activated to ramified (resting state). (**D**) Vortioxetine induces dendritic arborization, increasing levels of BDNF and stimulating Trk-B survival pathways. (**E**) Vortioxetine induces beta-amyloid plaque clearance in the CNS, improving synaptic plasticity and ameliorating recovery from temporary cognitive impairment. **Abbreviations**: EAE (experimental autoimmune encephalomyelitis); RAVLT (Rey Auditory Verbal Learning Test); TMT-A, TMT-B (Trail Making Test-A and B); SRT (Simple Reaction Time Task); CRT (Choice Reaction Time Task); BDNF (Brain-derived neurotrophic factor); TrkB (Tropomyosin receptor kinase B); LTP (Long-term potentiation).

Several studies showed that immunomodulation, mitigating central neuroinflammation, could impact mood disorders. Immunomodulatory strategies, also including regular physical exercise, exert positive effects on neuroinflammation in MS, reducing BDI cumulative scores and levels of neuroinflammatory mediators [58] (Fig. **2A**). Vortioxetine administration in mice with experimental autoimmune encephalomyelitis (EAE), a reliable model of MS, ameliorated the anxious and depressive phenotype of these mice, suggesting an action on central neuroinflammation [59] (Fig. **2B**). Vortioxetine was also able to interfere with the neurotoxic effects of microglial cells, favoring the maintenance of microglial cells in their resting state and reducing at the same time neuronal impairment caused by microglial activation [60]. Moreover, vortioxetine seems to influence central neuroinflammation acting on the microglial dynamic shift from M1 to M2 state in animal models, contributing to mitigate LPS-induced memory impairment by increasing IL-4 concentration in hippocampal subfields [61] (Fig. **2C**). Vortioxetine also promotes neuronal dendritic arborization, protecting astrocytes from injury [60] (Fig. **2D**). Interestingly, since all these effects are shared by many DMTs approved for the treatment of MS, such as dimethyl fumarate [62], fingolimod [63], siponimod [64], and ozanimod [65], vortioxetine could similarly act against the neurodegeneration processes in MS, representing an ideal add-on therapy to conventional DMTs.

## 6 ROLE OF VORTIOXETINE ON SYNAPTIC PLASTICITY: FROM ANIMAL MODELS TO HUMANS

Synaptic dysfunction and loss have been recently identified as key components of the inflammatory neurodegenerative damage occurring in EAE and MS brains [12]. Besides their effects of enhancing monoaminergic synaptic transmission, vortioxetine induces changes in intracellular signaling pathways, key gene expression, and neural plasticity presumably related to its specific efficacy. Administration of vortioxetine (but not of fluoxetine or ketamine) transiently increased the expression of several genes involved in neuroplasticity [66], and the same molecule, contrary to fluoxetine, promoted early changes in dendritic branches and synaptic structure in the rat hippocampus [67]. A significant part of this effect could be mediated by BDNF, which is involved in the modulation of inflammation and neurodegeneration in MS [68] by increasing its levels through the TrkB pathway [67] (Fig. **2D**). Importantly, MS patients with low CSF levels of beta-amyloid resembled the typical AD alterations, with a significant impairment of synaptic plasticity reserve [69]. In experimental models, vortioxetine was found to be able to correct beta-amyloid brain deposits in a model of chronic depression [70]. Moreover, a recent study showed significantly higher levels of serum BDNF in vortioxetine-treated subjects than in escitalopram-treated patients [71], in agreement with the idea that vortioxetine could positively impact synaptic plasticity reserve in MS patients by interfering with beta-amyloid metabolism and favoring the synaptic effect of BDNF (Fig. **2E**). Finally, it was demonstrated an association between serum BDNF levels and the amelioration of cognitive abilities in patients treated with vortioxetine compared to escitalopram, emphasizing the involvement of this neurotrophin in cellular effects mediated by the drug [72].

## CONCLUSION

Vortioxetine represents an innovative new treatment option for depression, combining high efficacy with a specific effect on cognitive symptoms and anhedonia, potentially contributing to relieving the impact of disability accumulation in depressed patients with MS [73, 74]. It is well known from the literature that the use of vortioxetine could be avoided in the case of specific psychiatric comorbidities, such as bipolar depression [75] and generalized anxiety [76], for which the use of traditional SSRIs and SNRIs should be preferred.

Although clinical trials are needed to confirm these aspects, due to its emerging central anti-inflammatory and neuroprotective effects, vortioxetine appears as a potential disease-modifying agent, able to complement the effects of the approved DMTs in relapsing-remitting and progressive MS patients.

## AUTHORS’ CONTRIBUTIONS

The authors confirm contribution to the paper as follows: study conception and design: E.D., P.A., M.C., E.C., A.C., G.A.M., M.S., V.T., V.T.C., R.T., D.C: conceptualization; E.D. and D.C., writing; E.D., A.C., M.S., V.T., A.B., D.C.: revision. All authors reviewed the results and approved the final version of the manuscript.

## Figures and Tables

**Table 1:** Role of vortioxetine: from preclinical and pharmacological evidence to clinical applications.

**Paper**	**Study Design**	**Results**
Sanchez *et al*., 2015	Review of preclinical and clinical data on vortioxetine	Specific antagonism for (HT) receptors 5-HT3, 5-HT1D and 5-HT7, partial agonism for 5-HT1B, and agonism for 5-HT1A Modulation of NE, dopamine, acetylcholine, histamine, glutamate, and GABA
Baune *et al*., 2018	Network meta-analysis	Effects of vortioxetine on digit symbol substitution test (DSST) rather than other SSRIs and SNRIs
Adamo *et al*., 2021	Review of clinical data	Vortioxetine 5-HT3 postsynaptic antagonism (reduction of hyperalgesia) and 5-HT7 postsynaptic antagonism (increased analgesia)
Santos García *et al*., 2022	Open-Label Prospective Study	Administration of vortioxetine causes amelioration of anxiety and depressive symptoms in patients with Parkinson's disease
Padovani *et al*., 2023	Delphi consensus	Administration of vortioxetine causes amelioration of anxiety and depressive symptoms in patients with Alzheimer's disease
Gil-Sanchez *et al*., 2024	Case series study	Administration of vortioxetine causes amelioration of anxiety and life quality attention. No amelioration for information processing and fatigue
McIntyre *et al*., 2014 Mahableshwarkar *et al*., 2015	Randomized placebo-control double-masked study	Vortioxetine causes amelioration of cognitive symptoms in patients with MDD.
Talmon *et al*., 2014	Preclinical study	Anti-inflammatory and immunomodulatory effects (M1-M2 shift) of vortioxetine in human monocytes/macrophages
du Jardin *et al*., 2016	Preclinical study	Increased synaptic plasticity in rat frontal cortex after vortioxetine administration
Chen *et al*., 2016	Preclinical study	Increased dendritic arborization and plasticity in rat hippocampus after Vortioxetine administration

**Note**: The Table summarizes the most critical and relevant papers on vortioxetine cited in this perspective article. **Abbreviations**: 5-HT (5-hydroxytryptamine); NE (norepinephrine); GABA (gamma-aminobutyric acid); MDD (major depressive disorder).

## LIST OF ABBREVIATIONS

5-HT3: 5-hydroxytryptamine-3
AD: Alzheimer’s Disease
BDI: Beck Depression Inventory
BDNF: Brain-derived Neurotrophic Factor
CSF: Cerebrospinal Fluid
DMTs: Disease-modifying Treatments
DSST: Digit Symbol Substitution Test
EAE: Experimental Autoimmune Encephalomyelitis
EDSS: Expanded Disability Status Scale
GABA: Gamma-aminobutyric Acid
MDD: Major Depressive Disorder
MEI: Montgomery and the Motivation and Energy Inventory
MRI: Magnetic Resonance Imaging
MS: Multiple Sclerosis
P-MS: Progressive MS
PD: Parkinson’s Disease
RAVLT: Rey Auditory Verbal Learning Test
RR-MS: Relapsing-remitting MS
SNRIs: Selective Noradrenaline Reuptake Inhibitors
SSRIs: Selective Serotonin Reuptake Inhibitors
STAI: State-trait Anxiety Inventory
STs: Symptomatic Therapies
TrkB: Tropomyosin Receptor Kinase B
